# Variability of systemic and oro-dental phenotype in two families with non-lethal Raine syndrome with *FAM20C* mutations

**DOI:** 10.1186/s12881-015-0154-5

**Published:** 2015-02-21

**Authors:** Ana Carolina Acevedo, James A Poulter, Priscila Gomes Alves, Caroline Lourenço de Lima, Luiz Claudio Castro, Paulo Marcio Yamaguti, Lilian M Paula, David A Parry, Clare V Logan, Claire E L Smith, Colin A Johnson, Chris F Inglehearn, Alan J Mighell

**Affiliations:** Oral Care Center for Inherited Diseases, University Hospital of Brasilia, Department of Dentistry, Health Sciences School, University of Brasilia, Brasilia, Brazil; Section of Ophthalmology and Neuroscience, University of Leeds, Leeds, UK; Department of Pediatrics, School of Medicine, University of Brasilia, Brasilia, Brazil; Section of Genetics, School of Medicine, University of Leeds, Leeds, UK; Department of Oral Medicine, School of Dentistry, University of Leeds, Leeds, UK

**Keywords:** Raine syndrome, *FAM20C*, Amelogenesis imperfecta, Dentine, Bone mineralization, Ectopic mineralisation

## Abstract

**Background:**

Raine syndrome (RS) is a rare autosomal recessive bone dysplasia typified by osteosclerosis and dysmorphic facies due to *FAM20C* mutations. Initially reported as lethal in infancy, survival is possible into adulthood. We describe the molecular analysis and clinical phenotypes of five individuals from two consanguineous Brazilian families with attenuated Raine Syndrome with previously unreported features.

**Methods:**

The medical and dental clinical records were reviewed. Extracted deciduous and permanent teeth as well as oral soft tissues were analysed. Whole exome sequencing was undertaken and *FAM20C* cDNA sequenced in family 1.

**Results:**

Family 1 included 3 siblings with hypoplastic Amelogenesis Imperfecta (AI) (inherited abnormal dental enamel formation). Mild facial dysmorphism was noted in the absence of other obvious skeletal or growth abnormalities. A mild hypophosphataemia and soft tissue ectopic mineralization were present. A homozygous *FAM20C* donor splice site mutation (c.784 + 5 g > c) was identified which led to abnormal cDNA sequence. Family 2 included 2 siblings with hypoplastic AI and tooth dentine abnormalities as part of a more obvious syndrome with facial dysmorphism. There was hypophosphataemia, soft tissue ectopic mineralization, but no osteosclerosis. A homozygous missense mutation in *FAM20C* (c.1487C > T; p.P496L) was identified.

**Conclusions:**

The clinical phenotype of non-lethal Raine Syndrome is more variable, including between affected siblings, than previously described and an adverse impact on bone growth and health may not be a prominent feature. By contrast, a profound failure of dental enamel formation leading to a distinctive hypoplastic AI in all teeth should alert clinicians to the possibility of *FAM20C* mutations.

**Electronic supplementary material:**

The online version of this article (doi:10.1186/s12881-015-0154-5) contains supplementary material, which is available to authorized users.

## Background

Raine syndrome (OMIM #259775), first described in 1989, is a rare inherited disorder (prevalence estimated at < 1/1,000,000 [1]) characterized primarily by osteosclerotic bone dysplasia due to dysregulation of bone metabolism with ectopic soft tissue mineralization [2]. It is typically evident at birth and can result in death in infancy. The first report was of a female neonate who died shortly after vaginal delivery, where the father was believed to also be the mother’s father. The neonate had osteosclerosis, microcephaly, exophthalmos, nasal and midface hypoplasia, gingival hyperplasia, cleft palate and low-set ears [2]. The clinical phenotype of Raine syndrome was further defined by descriptions of other cases lethal in infancy [3-12]. Recessive mutations in *FAM20C* have since been identified as the cause of Raine syndrome [13]. The identification of additional families has allowed the clinical phenotype associated with *FAM20C* mutations to broaden with new features still being described [1].

More latterly, it has been recognized that Raine syndrome is not always lethal and survival into adulthood is possible [14-18]. Accordingly, disease due to *FAM20C* mutations may be under-recognised with a higher prevalence than that described for Raine Syndrome. Generalized skeletal osteoscelerosis and associated consequences remain a prominent feature although the impact on bone growth and development covers a broad spectrum. There is variable craniofacial dysmorphism that is most pronounced in lethal cases. Ectopic mineralization has been reported at different sites including the brain and kidneys in both lethal and non-lethal cases [3,5,15,19-22]. Dental abnormalities include Amelogenesis Imperfecta (AI – inherited developmental enamel formation abnormalities) and dentine dysplasia, but these have not been characterized in detail [15,16].

The wide clinical phenotype spectrum of the syndrome has broadened further with description of childhood hypophosphatemia consequent to renal phosphate wasting with correction of the hyperphosphaturia to near normal levels in adulthood [14-16]. Paradoxically, children with *FAM20C* mutations have osteosclerosis rather than rickets, which is the typical association with hyperphosphaturia-induced hypophosphatemia. This insight has contributed to the understanding of the spectrum of clinical diseases that share raised FGF23 levels as a common feature.

FAM20C is an evolutionarily conserved Golgi casein-kinase that phosphorylates several secretory calcium binding–phosphoproteins (SCPP) involved in biomineralisation, including the small integrin-binding ligand and N-linked glycoproteins or SIBLINGs [23-25]. The phosphorylation state of this wide range of proteins influences biomineralisation in health, and when dysregulated, contributes to mineralised tissue diseases and ectopic mineralisation [26,27]. FAM20C directly phosphorylates the fibroblast growth factor 23 (FGF23), inhibiting its glycosylation and results in FGF23 proteolysis and inactivation [25]. FGF23 is an important phosphate homeostasis regulator, once it controls its renal resorption process, acting as a phosphaturic agent. *Fam20C* knock-out mouse models are characterised by disruption of multiple cellular pathways involved in bone and tooth development [28,29].

To date, 18 *FAM20C* mutations, including whole gene deletion, microdeletion, homozygous or compound heterozygous missense and splice-site mutations have been reported [13-18]. The aim of the present study was to characterize the systemic, oro-dental features and causative genetic mutations of two consanguineous Brazilian families with multiple family members who have survived into adolescence and adulthood with ill-defined syndromes. Genetic analysis of both families revealed mutations in *FAM20C*, confirming previous reports that individuals with mutations in FAM20C may survive beyond infancy. Furthermore, observation of the characteristic dental phenotype should prioritise *FAM20C* as the cause of disease.

## Methods

This study was approved by the Ethics Board of the Brazilian Ministry of Health-CONEP and informed consent was obtained from all participants. Written informed consent was obtained from the patients and/or their parents for publication of this article and any accompanying images. A copy of the written consent is available for review by the Editor of this journal. Affected family members from 2 families were referred to the Oral Care Center for Inherited Diseases of the University Hospital of the University of Brasilia, Brazil. Physical and dental examinations were performed on eight members of Family 1 and six from Family 2. Biochemical blood analyses, limb radiographs and skull computed tomography results were obtained from medical records. Incisional biopsies of dental periapical lesions, pericoronal tissues of unerupted teeth and gingiva were performed; specimens were fixed with 10% buffered formalin, embedded in paraffin, stained with Haemotoxylin and Eosin and examined by light microscopy (Axiophot, Zeiss). Cross sections of demineralized teeth, as well as ground sections of deciduous and permanent teeth, extracted by therapeutic indication, were also examined with light microscopy (Axiophot, Zeiss).

Genomic DNA samples from Family 1 (individuals IV:4, IV:5 and IV:6) were genotyped using Affymetrix 5.0 SNP microarrays. Whole exome sequencing was performed using the SureSelect All Exon 50 Mb reagent. Briefly, 3 μg of genomic DNA from patient IV:5 was processed according to the Agilent SureSelect Library Prep protocol. Sequencing was performed using an 80 bp single-end protocol on an Illumina GAIIx sequencer. Resulting sequence reads were aligned to the human (hg19) genome reference sequence using Novalign software and processed in the SAM/BAM format using Picard, SAMtools and the Genome Analysis Toolkit (GATK) java programs [30]. Variants were called in the VCF format according to standard GATK protocols and filtered using vcfhacks (http://vcfhacks.sourceforge.net/). RNA from affected individuals was collected using the Tempus Blood RNA kit (Life Technologies, Carlsbad, USA). First strand cDNA synthesis was performed using M-MLV reverse transcriptase (Life Technologies) according to the manufacturer’s instructions. PCR amplification of coding and flanking intronic regions was performed in both families using HotShot Diamond Mastermix (Clent Life Sciences, UK) according to the manufacturer’s standard conditions. The PCR reaction mixture was initially denatured for 5 minutes at 95°C, followed by 35 cycles of a further denaturing step at 94°C for 30 seconds, an annealing stage for 30 seconds at 60°C, and an extension phase at 72°C for 30 seconds. A final extension step at 72°C for 10 minutes was performed. PCR products were checked on a 2.5% agarose gel. Prior to performing sequencing reactions, PCR products were purified using ExoSAP-IT exonuclease (USB Corporation, Cleveland, USA) according to the manufacturer’s instructions. Purified PCR products were sequenced using the BigDye Terminator Kit v.3.1 (Applied Biosystems). Following amplification, sequencing products were ethanol precipitated by standard protocols and re-suspended in 10 μl of Hi-Di formamide (Applied Biosystems). Samples were run on an ABI 3130XL Genetic Analyser using polymer POP-7 and the default RapidSeq36POP7 module and the resulting data analysed using SeqScape version 2.5 (Applied Biosystems).

## Results

**Figure 1:**
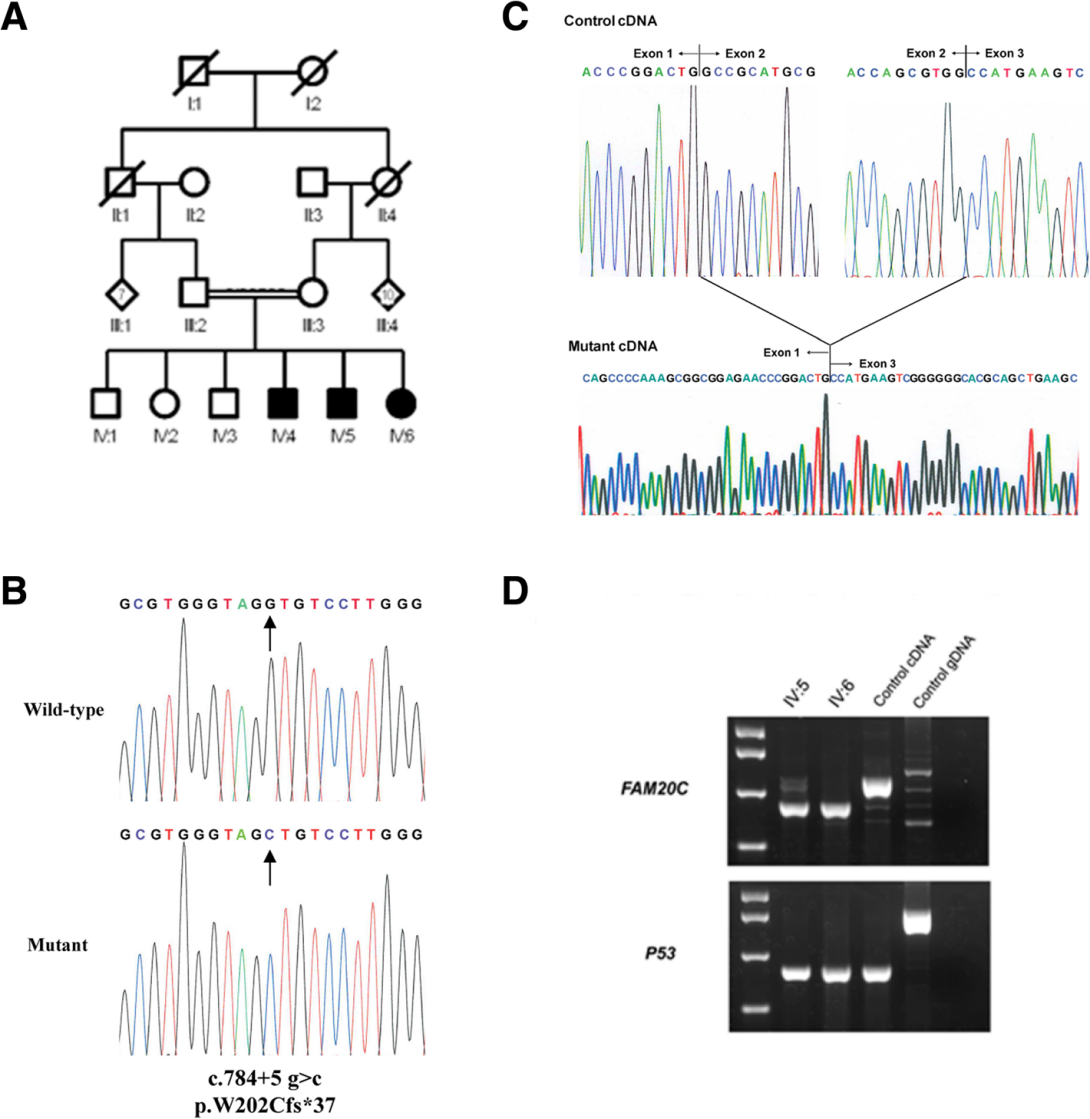
**Pedigree and genetic results from Family 1. (A)** Pedigree of the family I suggesting an autosomal recessive mode of inheritance. **(B)** Electropherogram showing wild-type and mutant sequence of the p.W202Cfs*37 mutation identified in Family 1. **(C)** Analysis of the cDNA from affected family members revealed the mutation resulted in skipping of exon 2. **(D)** RT-PCR over the mutation shows the reduction in transcript size in the mutant allele and also the presence of some wild-type sized transcript.

**Figure 2:**
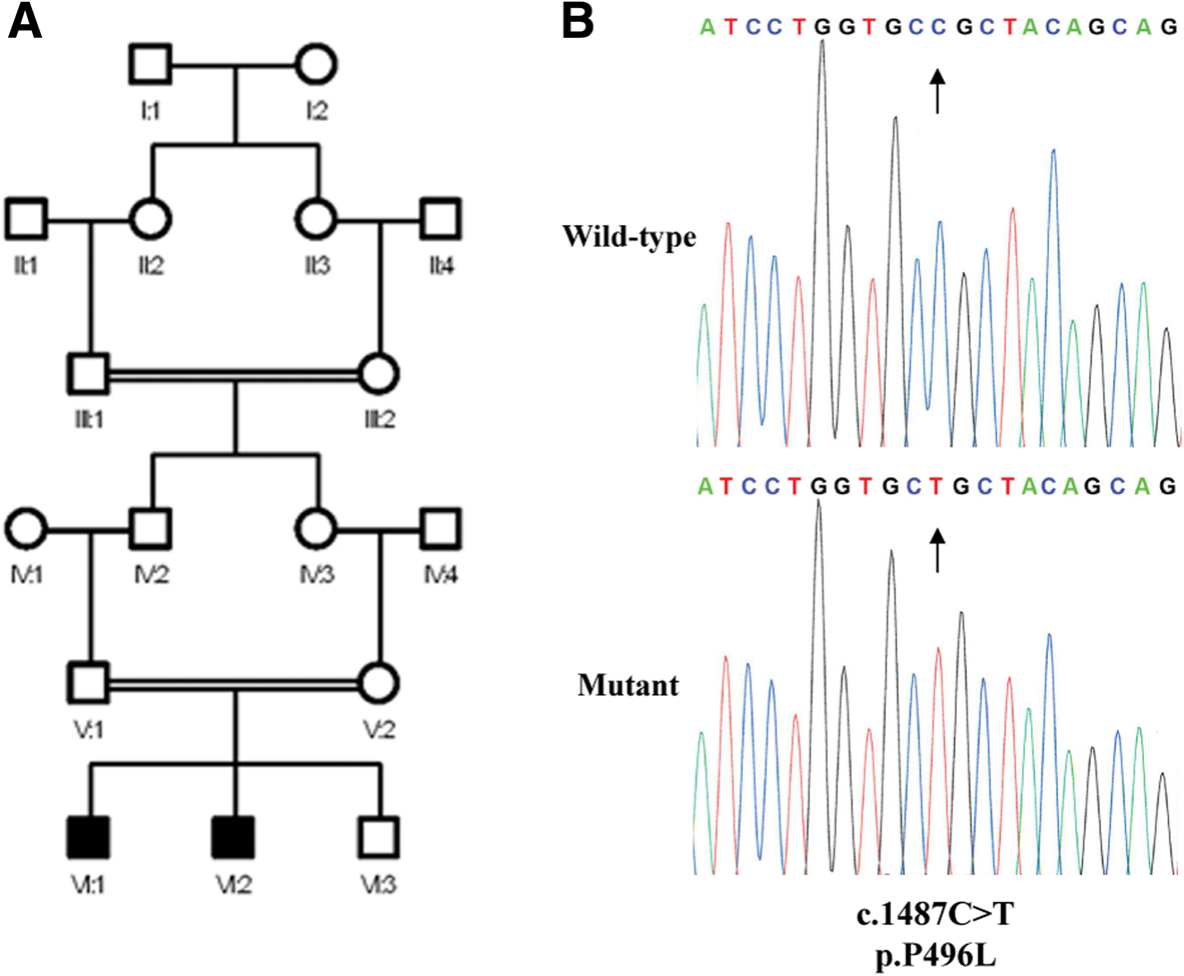
**Pedigree and genetic analysis of Family 2. (A)** Pedigree of family II suggesting an autosomal recessive mode of inheritance. **(B)** Electropherograms showing the wild-type and mutant alleles for the p.P496L mutation identified in Family 2.

The two unrelated Brazilian families reported in this study were initially diagnosed with autosomal recessive AI as part of unrecognized syndromes. Both families reported consanguineous marriages (Figures 1A & 2A). Comparisons of the results of biochemical analyses, together with systemic and oro-dental features of affected individuals are summarized in Tables 1, 2 and 3.

**Table 1:** **Summary of systemic features in non-lethal Raine syndrome individuals**

**Family**	**Family 1**			**Family 2**	
**Pedigree number**	**IV-4**	**IV-5**	**IV-6**	**VI-1**	**VI-2**
**Sex**	M	M	F	M	M
**Age at last review (yrs)**	27	22	21	13	12
**Neurological**					
Intracranial calcifications	NA	NA	+	+	+
Seizures	+	-	NA	+	-
**Craniofacial**					
Skull osteosclerosis	-	-	-	-	-
Microcephaly	+	+	-	+	+
Craniosynostosis	-	-	-	+	+
Exophthalmos	+	+	+	+	+
Visual Impairment	-	-	-	+	-
Midface hypoplasia	+	+	+	+	+
Depressed nasal bridge	-	-	-	+	+
Choanal atresia	+	-	-	+	-
Low set ears	-	-	-	+	+
Dysplastic ears	+	+	+	-	-
Hearing loss	+	+	+	-	-
**Skeletal**					
Short stature	-	-	-	+	+
Short limbs	-	-	-	+	+
Undermineralized long bones	-	-	-	+	+
Short fingers	-	+	-	+	+
Clinodactyly	-	-	-	+	+
**Genito-urinary**					
Renal calcifications	-	-	-	+	-

+: present; −: not present; NA not assessed.

**Table 2:** **Summary of biochemical analyses in non-lethal Raine syndrome individuals**

**Patient (Family.)**	**Family 2**					**Family 2**					
**Pedigree number**	**IV-4**		**IV-5**	**IV-6**		**VI-1**			**VI-2**		
Gender	Male		Male	Female		Male			Male		
Age (years)	16	24	15	13	18	7	9	11	6	8	10
Serum calcium (8.8 – 11 mg/dL)	9.1 (N)	9.3 (N)	9.6 (N)	10,2 (N)	9.0 (N)	8.9 (N)	9.2 (N)	9.2 (N)	8.7 (N)	9.6 (N)	9.0 (N)
Serum phosphate (age dependent)	4.9 (N)	2.3 (↓)	4.6 (N)	4.7 (N)	2.6 (↓)	4.7 (N)	3.8 (N)	3.3 (↓)	4.4 (N)	3.4 (↓)	3.3 (↓)
Serum alkaline phosphatase (age dependent)	1462 (↑)	90 (N)	432 (↑)	380 (↑)	89 (N)	406 (↑)	457 (↑)	--	365 (↑)	385 (↑)	--
PTH (12 – 65 pg/mL)	NA	NA	NA	NA	NA	95.7 (↑)	55 (N)	71.4 (↑)	103 (↑)	35.8 (N)	52 (N)
Urinary calcium (<4 mg/kg/day)	--	--	79.8	112,2	--	0.5 (N)	2.1 (N)	0.71 (N)	0.8 (N)	1.3 (N)	0.65 (N)
Urinary phosphate (<15 mg/kg/day)	--	--	122.3	143,6--	--	11.31 (N)	23.2 (↑)	15.7 (↑)	10.5 (N)	30.5 (↑)	11.0 (N)
TPR (>85%)	--	--	--	--	--	89 (N)	88 (N)	79 (↓)	90.5 (N)	86 (N)	84 (↓)

N = normal; NA = not assessed. Age-dependent metabolites: Serum phosphate: < 6 months: 4,8 – 7,4 mg/dL; 6 m – 5 years: 4,5 - 6,2 mg/dL;6 y – 1y before growth spurt: 3,6 – 5,8 mg/dL; after growth spurt: 2,5 – 4,5 mg/dL Alkaline phosphatase: 7 months −1 years: < 462 U/L; 1 – 3 years: < 281 U/L; 4–6 years: < 269 U/L; 7–12 years:< 300 U/L; 13–20 years: men: < 390 U/L, women: < 187 U/L; adults: men: 40–129 U/L, women: 35–104 U/L. Convertion: pmol/L x 9.497 = pg/mL; mmol/L x 18 = mg/dL.

**Table 3:** **Summary of oro-dental features in non-lethal Raine syndrome individuals**

**Family**	**Family 1**			**Family 2**	
**Pedigree number**	**IV-4**	**IV-5**	**IV-6**	**VI-1**	**VI-2**
Micrognathia	**-**	**-**	**-**	**-**	**-**
High palate	**+**	**+**	**+**	**+**	**+**
Malocclusion	**+**	**+**	**+**	**+**	**+**
Gingival enlargement	**+**	**+**	**+**	**+**	**+**
Gingival and/or follicular calcifications	**+**	**+**	**+**	**+**	**+**
Amelogenesis Imperfecta	**+**	**+**	**+**	**+**	**+**
Incisal notch of central incisors	**+**	**+**	**-**	**+**	**+**
Interglobular dentine	**+**	**+**	**+**	**+**	**+**
Ectopic eruption of upper premolars	**-**	**+**	**+**	**+**	**-**
Unerupted permanent teeth	**-**	**-**	**-**	**+**	**+**
Pulpal calcifications	**-**	**+**	**+**	**-**	**-**
Incomplete root formation	**-**	**-**	**-**	**+**	**+**
Periapical lesions	**+**	**+**	**+**	**+**	**+**

+: present; −: not present.

### Family 1

**Figure 3:**
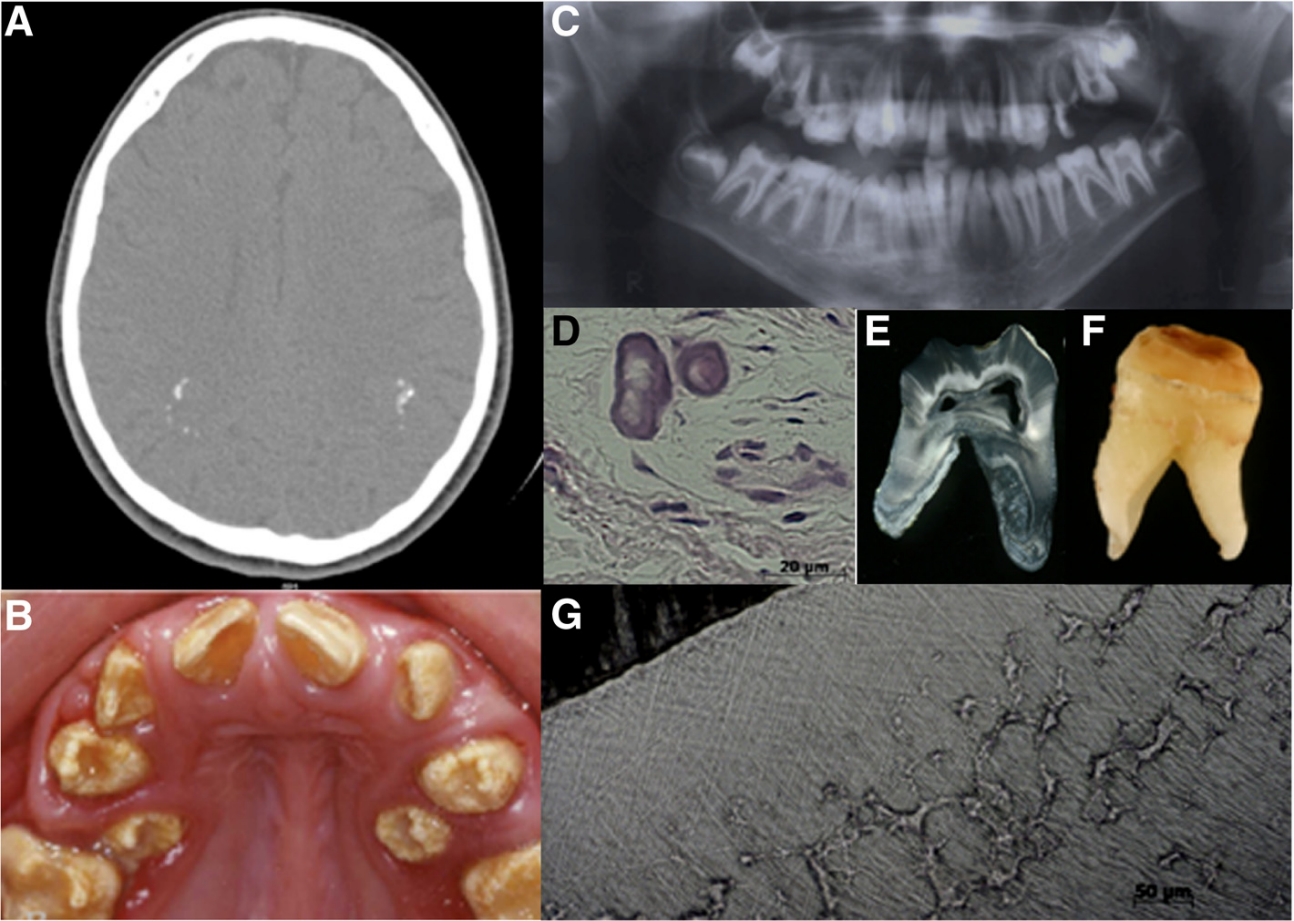
**Radiographic and oro-dental features of Family 1. (A)** Computerized tomography (CT) scan revealed intracranial calcifications in the parieto-occipital region in patient IV-6. **(B)** Clinical photograph of patient IV-4 showing alterations in the tooth shape, reduction of the enamel thickness and yellow and brown discoloration. Note the rough and pitted surface of enamel. Incisal notches were observed in the central incisors. **(C)** Dental radiographs illustrated absence of the normal, differential radiodensity between enamel and dentine, whilst periapical radiolucency’s and associated alveolar bone loss were consistent with loss of dental pulp vitality and associated periodontal involvement. **(D)** The histopathological analysis of the alveolar gingival tissue showed ectopic calcifications. **(E)** Macroscopic and **(F)** ground sections of an extracted left first molar revealed severe hypoplastic enamel with surface pitting. **(G)** Interglobular dentine of the circumpulpar dentine was observed, alongside normal dentine in some areas.

A 16 year old male patient (IV: 4) was referred to the Oral Care Center for Inherited Diseases of the University Hospital of the University of Brasilia, Brazil with autosomal recessive AI. The parents were first cousins and reported that 3 of their 6 children, the proband, an additional male aged 12 years (IV:5) and a female aged 10 years (IV:6), had similar, abnormal dental problems that contrasted with the teeth of their other children (Figure 3). All six siblings were examined as well as the mother and a first cousin, confirming the presence of AI associated with an unknown syndrome. The initial clinical and dental examination of the affected siblings was in 2003. They have since received dental treatment and have been followed up since then.

The mother reported uncomplicated pregnancies for the affected siblings. Individual IV-4 had perinatal respiratory problems due to choanal atresia that was surgically corrected. Mildly dysmorphic facies were observed in the three affected siblings as they grew (Table 1). By late teens microcephaly was recognised in IV-4. There were no other evident skeletal abnormalities evident in the affected siblings.

No developmental psychomotor delay was present. Individual IV-4 started with seizures at 10 years of age and anticonvulsant medication was initiated. The only affected individual who agreed to perform a brain computerized tomography (CT) scan was IV-6, which revealed intracranial calcifications (Figure 3A; Table 1).

A variable spectrum of hearing impairment was evident in the affected individuals. Severe conductive hearing loss leading to left-sided deafness was present in patient IV-6. Mild sensorineural hearing loss was present in IV-4, IV-5 and the mother, but investigations to fully characterise the abnormality were incomplete (Table 1). Mastoid CT imaging of IV-4 and IV-5 were normal.

Renal ultrasounds of all the three affected siblings were within normal limits with no nephrolithiasis or nephrocalcinosis. Serum phosphate levels were mildly decreased in IV-4 and IV-6 at the ages of 24 and 18 years respectively (Table 2).

The affected individuals had histories of recurrent periapical abscesses in both dentitions. The initial examination of the three siblings identified a shared phenotype of midface hypoplasia, micrognathia, a high arched and narrow palate, open bite malocclusion and abnormal enamel (Figure 3C; Table 3). Very poor oral hygiene was observed in all the affected siblings with abundant dental plaque, associated severe gingivitis, calculus and dental caries.

All erupted permanent teeth displayed a yellow brownish discoloration. The mother reported that the deciduous teeth were small and yellow. In IV-4 and IV-5, incisal notches were observed in the central incisors. Dental enamel of all the permanent teeth was similar in the affected siblings with poorly mineralised hypoplastic enamel that was easily removed with dental hand instruments. Enamel pitting was observed in several teeth. No eruption delay was observed, but there was ectopic eruption of premolars, probably due to the micrognathia and consequent lack of space (Figure 3B).

The dental radiographic examination showed complete permanent dentitions with well-developed roots. However, no difference in radiodensity was observed between enamel and dentine. In IV-6 pulpal calcification was present in lower posterior teeth. Lateral and periapical radiolucencies were observed in all affected siblings, which was consistent with periapical and periodontal abscesses (Figure 3C).

Histopathological analysis of pericoronal soft tissues of unerupted third molars revealed ectopic calcification (Figure 3D). The ground sections of permanent teeth showed a thin enamel layer. In focal regions of some but not all teeth a histological pattern of interglobular dentine was observed (Figure 3E-G). Mantle dentine appeared normal. A lack of fusion of the mineralization foci, also called calcospherites, was observed in circumpulpal crown and root dentine, leaving regions of hypomineralised matrix (Figure 3G).

Cytogenetic analyses did not reveal any chromosomal alterations. SNP array analysis in family 1 revealed 4 regions of homozygosity shared between the three affected individuals, these being on chromosomes 7 (1–2,547,185 bp), 9 (111,978,486-117,000,801 bp), 11 (119,214,798-131,456,204 bp) and 13 (109,495,367-115,086,044 bp). Exome sequencing revealed four potentially functional sequence variants in the shared homozygous regions following removal of variants with a minor allele frequency (MAF) of 1% or higher in dbSNP138, NHLBI exome variant server (EVS) or locally sequenced exomes. A missense change in *COL27A1* (NM_032888:c.370C > G, p.L124V) was discounted as it is present in dbSNP 138 (rs149948860) with a MAF of 0.3%, in the EVS database with a frequency of 0.8% in African Americans, and is predicted to be benign by 3/6 mutation testing packages (Additional file 1: Table S1). A missense variant in *ROBO3* (NM_022370:c.1838C > T, p.T613I) was also present in dbSNP 138 (rs200202857) although with no known allele frequency, but has a frequency of 0.9% on the EVS database, and is predicted to be benign by 4/6 mutation testing packages (Additional file 1: Table S1). Furthermore, homozygous mutations in *ROBO3* have been identified as a cause of gaze palsy with progressive scoliosis (MIM 607313), a distinctly different phenotype to that described here, so this variant was again considered unlikely to be the pathogenic mutation in this family. A missense change in *KIRREL3* (NM_032531:c.1007G > A, p.R336Q) was present in dbSNP 138 (rs114378922) and EVS with a MAF of 0.3% in African Americans, was again predicted to be benign in 3/6 mutation prediction packages (Additional file 1: Table S1) and was therefore assumed to be a rare polymorphism.

The remaining variant lies in the splice donor consensus sequence after exon 2 of *FAM20C* (NM_020223; c.784 + 5 g > c; Figure 1B). This variant segregated with the disease phenotype, is neither present in dbSNP 138 nor the EVS database and was predicted by NetGene2 to reduce the splicing donor affinity from 0.98 to 0.60 [31]. Mutations in *FAM20C* cause Raine syndrome, and given the overlap between this condition and the phenotype observed in family 1, this variant was investigated further. cDNA from the blood of affected individuals was PCR amplified across the involved splice site. This analysis revealed skipping of *FAM20C* exon 2 in the cDNA (Figure 1C). This is predicted to cause a frameshift and premature stop codon (p.W202Cfs*37). Visualization of the cDNA amplification products by electrophoresis confirmed the predominance of a smaller product predicted from exon-skipping (Figure 1D) but also revealed low levels of a larger wild-type sized band for individual IV:5, raising the possibility that the c.784 + 5 g > c mutation creates a hypomorphic allele (Figure 1D). The *FAM20C* splicing change was therefore deemed to be the cause of the phenotype in this family.

### Family 2

**Figure 4:**
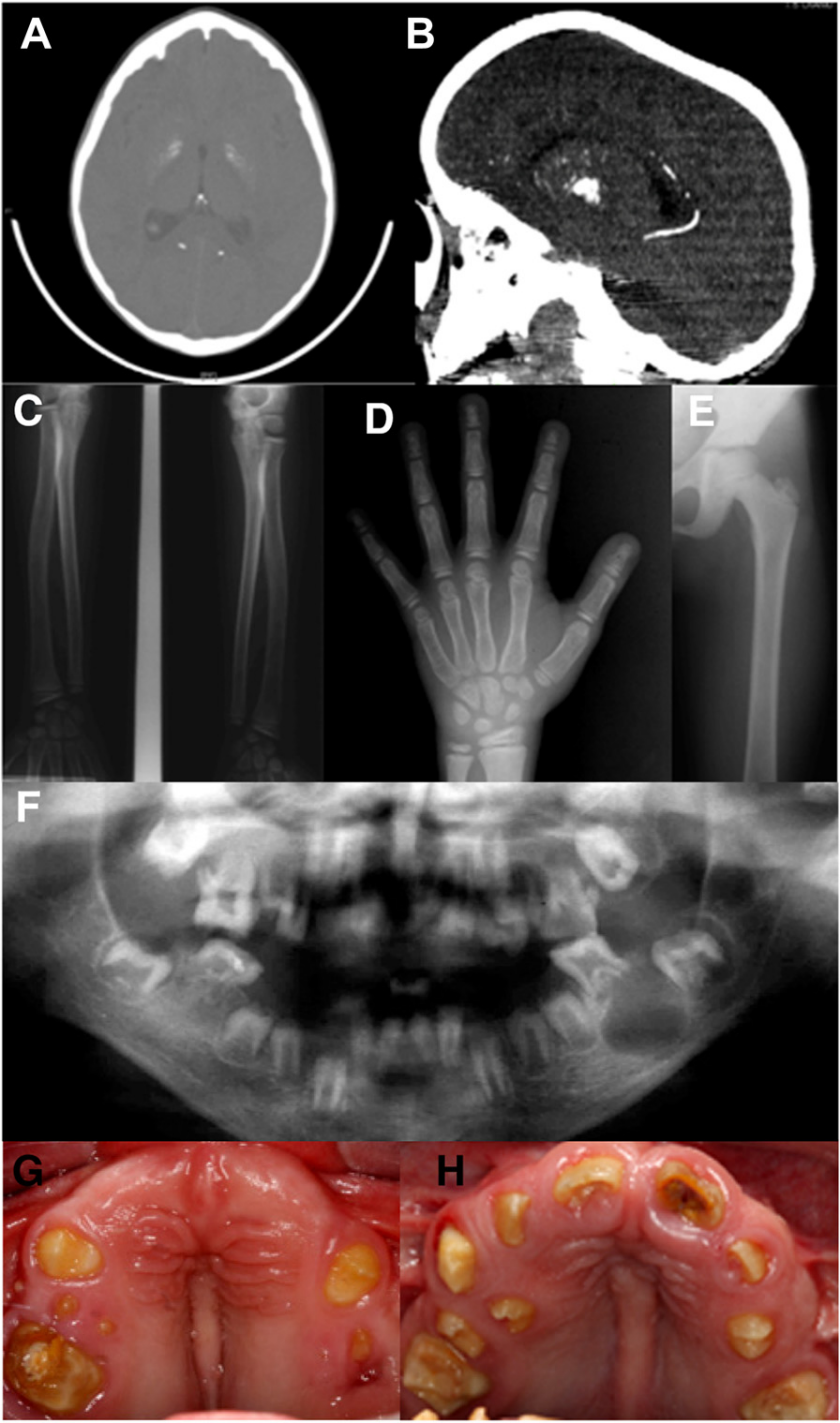
**Radiographic and oro-dental features of Family 2.** CT scanning identified **(A)** intracranial calcifications and **(B)** probably vascular calcifications in patient VI-1. Radiographs show **(C)** undermineralised long bones, **(D)** carpal bones and phalanges **(E)** and a mild radius bowing in patient VI-2. **(F)** Dental radiograph showing an absence of density differences between enamel and dentin, incomplete root formation and enlarged pulp chambers. Apical radiolucencies associated with permanent teeth are also present. **(G & H)** Affected family members presented permanent erupted teeth with yellow discoloration, hypomineralised and hypoplastic enamel. Severe delays in permanent tooth eruption were observed in both siblings.

Two male siblings (VI-1 and VI-2) born of first-degree cousins were referred by the Medical Genetics Clinic of the University Hospital of Brasilia at the ages of 5 and 4 years old with an unidentified syndrome. The parents revealed that the grandparents were also first cousins. The pregnancies of the two affected siblings were reported as uncomplicated. The older brother had respiratory problems at 1 month of age with choanal atresia diagnosed. Recurrent seizures started at that time and continued to occur. During the first six months he was hospitalized for different reasons without a definitive diagnosis. The younger brother was also hospitalized due to respiratory problems and recurrent pneumonia. Both brothers showed a delay in psychomotor development and started walking after two years of age. Both had microcephaly, low set ears, a hypoplastic nose with depressed nasal bridge, prominent alae nasae, down-slanting palpebral fissures and exophthalmos (Table 1). CT scanning identified intracranial calcification in both siblings when aged five and four years old, respectively. Both had craniosynostosis of the sagittal suture resulting in a scaphocephalic, saddle-shaped head. A further CT scan of VI-1 at age 11 years revealed probable intracranial vascular calcification (Figure 4A-B; Table 1). By this age he had developed severe visual impairment. An ophthalmological evaluation revealed nystagmus, retinal pigmentation, total blindness of the right eye and a left cataract compromising 90% of vision.

In both siblings, skeletal surveys showed small hands with bulbous fingertips and clinodactyly of the fifth finger. Radiographic examination revealed under-mineralized distal phalanges. Both had short stature and long bone radiographs revealed under-mineralization in the shaft and growth plate as well as bowing of the radius bones when 7 years old (Figure 4C-E; Table 1). There was no evidence of osteosclerosis in any of the investigated bones.

Biochemical evaluation demonstrated abnormalities at different time points with decreased serum phosphate, increased parathyroid hormone (PTH) and hyperphosphaturia (Table 2). The biochemical and radiographic findings were suggestive of hypophosphataemic rickets. Renal ultrasound was undertaken in VI-1 only and revealed microcalcification in the renal medullae (data not shown).

The oro-dental features of both siblings included midface hypoplasia, micrognathia, high arched and narrow palate, enlarged gingival and palatal mucosa, unerupted permanent teeth, hypoplastic AI (with very little enamel), and history of recurrent periapical dental abscesses in deciduous and permanent (Figure 4F-H; Table 3).

All erupted permanent dentition displayed a yellow brownish discoloration. In both brothers, an incisal notch of the central incisors was observed, even in the unerupted central incisors of the younger brother (Figure 4F & H). Dental enamel was similarly affected in both siblings and mainly characterised by hypoplasia, poor mineralisation and a rough appearance. The younger brother had a severe delay in permanent tooth eruption (Figure 4F). The radiographic examination revealed a complete permanent dentition with incomplete root formation, enlarged pulp chambers and root canals with impaired apex formation. Several unerupted permanent teeth had associated well-defined pericoronal radiolucencies. No density difference between enamel and dentine was observed. Periapical radiolucencies were observed in both siblings (Figure 4F; Table 3).

**Figure 5:**
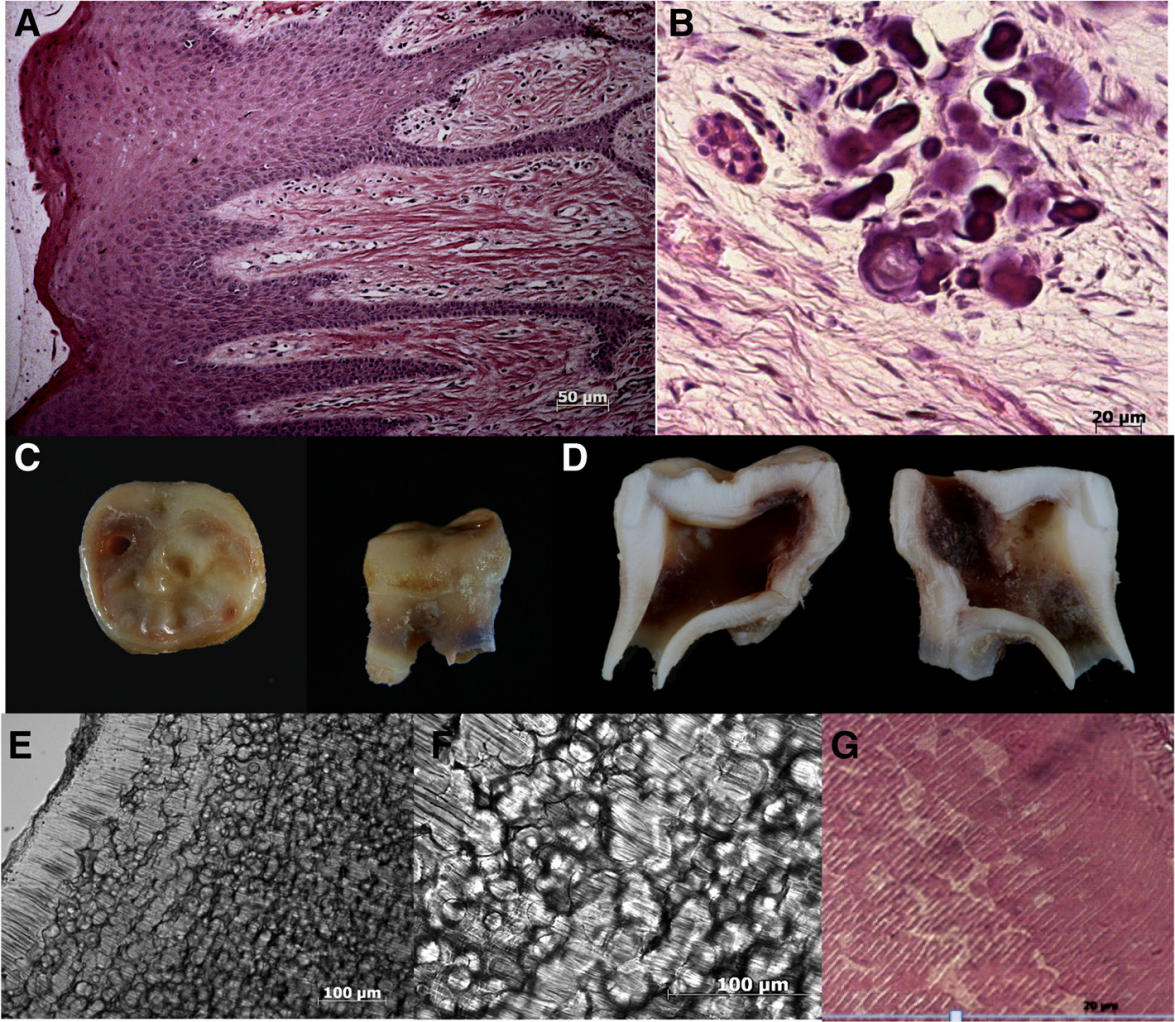
**Histopathological analyses of gingiva and teeth of Family 2. (A)** Histological analysis of patient gingiva revealed the presence of inflammatory infiltrate, epithelial acanthosis and gingival fibromatosis. **(B)** In pericoronal tissues, areas of ectopic calcifications were observed. **(C)** Analysis of first right molar of the patient VI-2 showed occlusal dental decay and incomplete root formation. **(D)** Sagittal median section of the teeth show large pulp chamber. **(E)** Ground sections reveal interglobular dentine except in the mantle dentine. **(F)** Severely affected circumpulpar dentine in increasing magnification. **(G)** Dentinal changes also observed in slides stained with HE after the same tooth demineralization.

Histopathological analysis of a gingival biopsy confirmed gingival hyperplasia and ectopic calcification was evident in the pericoronal tissues of unerupted teeth (Figures 5A-B).

The ground sections of permanent teeth showed a very thin enamel layer. Demineralized and ground sections showed mantle dentine that appeared normal and severely affected circumpulpal dentine had an interglobular appearance in the thin layer of crown and root dentine (Figure 5E-G). All examined teeth had interglobular dentine.

The careful phenotyping of affected individuals in this family suggested a non-lethal form of Raine syndrome as a diagnosis. The coding exons and flanking intronic sequence of *FAM20C* were therefore sequenced. This revealed two potentially pathogenic homozygous missense variants. The first was c.1672C > T (p.R558W) previously published as the cause of a lethal form of Raine syndrome [32]. However, this variant was not predicted damaging by various bioinformatic prediction softwares and is now present in dbSNP (rs62644536) with a MAF of 3.58% (n = 2256). This variant was therefore discounted as the cause of disease in this family. The second variant, c.1487C > T (Figure 2B) was found in the 9th exon leading to a missense substitution of a proline to a leucine (p.P496L). This variant is not present in dbSNP 138 or the >60,000 exomes of the Exome Aggregation Consortium (http://exac.broadinstitute.org/), and was excluded from a panel of 96 ethnically diverse control individuals and 15 Brazilian control individuals. Segregation of this variant in the family was consistent with it being the causative mutation. This change substitutes a residue that is fully conserved in both orthologues and paralogues (Additional file 1: Figure S1). Furthermore, P496 is a conserved feature of the putative activation loop within the C-terminal kinase domain of FAM20C (NCBI conserved domain cd10471) and was predicted to be pathogenic in all 6 of the bioinformatic prediction packages tested, suggesting the p.P496L mutation is likely to impair the kinase function of the protein.

## Discussion

A diagnosis of Raine Syndrome is triggered clinically by distinctive facial features and osteosclerosis with the potential for the condition to be lethal in early life [1]. Non-lethal Raine Syndrome has only been reported in a small number of individuals, with 1 compound heterozygous and 4 homozygous *FAM20C* mutations previously described [18]. *FAM20C* mutations affect many aspects of human biomineralisation. These can manifest as skeletal dysplasia that is more evident in the craniofacial than in vertebral and long bones, disrupted bone metabolism throughout life, inappropriate soft tissue mineralisation and abnormal tooth development. Nevertheless, the physiological role of FAM20C regarding the complex interplay between promoters and inhibitors of mineralization remains incompletely understood. The present study emphasizes the variability of this clinical phenotype, with the further expansion of the clinical and metabolic spectrum of non-lethal Raine Syndrome due to recessive *FAM20C* mutations, which may both be hypomorphic.

Family 1 was characterised by AI and mild facial dysmorphism as the presenting features. Whole exome sequencing identified a previously undescribed *FAM20C* homozygous splice site mutation, which on cDNA analysis disrupted splicing and the predicted translation. This led to the diagnosis of an attenuated form of non-lethal Raine syndrome. The presence of some wild-type cDNA on RT-PCR may explain the milder phenotype observed in this family, consistent with a hypomorphic mutation.

The mutation discovered in Family 1, plus literature review informed the molecular investigation of Family 2, in which clinical features including craniofacial dysmorphism and other clinical features suggested Raine syndrome. This was confirmed after detection of a previously unreported homozygous missense mutation in *FAM20C*. In common with several other cases of Raine Syndrome, the amino acid change in Family 2 is predicted to disrupt the CCD kinase domain that is essential to normal FAM20C function [1].

At the time of writing, affected individuals in both families have reached adolescence or early adulthood without life-threatening consequences, adding to the small number of individuals reported who have survived beyond infancy.

A generalised skeletal osteoscelerosis is the predominant bone phenotype described for patients with Raine Syndrome. Osteosclerosis was not identified in either of the studied families. Plain radiographs demonstrated that Family 2 individuals had under-mineralized bones at the time of investigation with radial bone bowing. Biochemical analyses were consistent with a hypophosphataemic rickets phenotype. *Fam20c* null mice also have features of hypophosphataemic rickets [33]. Hypophosphataemia is a prominent feature of other reported non-lethal cases with *FAM20C* mutations, but this has previously been paired with osteosclerosis [14-18]. Age at the time of investigation is likely to be an important factor and one that is poorly understood given the paucity of cases reported to date. A 61-year old Japanese man with hypophosphataemic osteomalacia attributed to homozygous *FAM20C* mutations highlights the current limited understanding [18] of the impact of FAM20C on bone turnover. This individual had leg bowing as a child and short stature as well as other clinical features consistent with abnormal bone metabolism. In contrast to the reported Japanese man and Family 2, there was no obvious clinical impact on bone turnover in Family 1. Biochemical investigations for a phosphate-wasting disorder have not been undertaken, although the family are aware of this possibility and of the need for ongoing long-term follow up.

Ectopic soft tissue calcifications have been reported previously in Raine syndrome [3,5,15]. Intracranial, renal and ectopic gingival calcifications were observed in both of the families described here, suggesting that this finding is a consistent component of Raine syndrome due to *FAM20C* mutations. This is further supported by Vogel *et al.* who reported vascular calcifications in Fam20c-null mice [28].

The detailed dental and tooth phenotyping reported here highlights and adds new information regarding the variable severity, the impact on both enamel and dentine formation and the similarities with dental changes in other phosphate-wasting conditions. Dental crown and root morphology alterations along with altered enamel and dentine indicate that *FAM20C* mutations impact on early and late odontogenesis. This is consistent with the fact that FAM20C is widely expressed in dental cells including in ameloblasts, odontoblasts, cementoblasts and periodontal ligament fibroblasts [34,35].

Enamel formation was severely affected in both families in a similar way, with a thin layer of poorly mineralized enamel clinically manifesting as hypoplastic AI. *Fam20c* knockout mice have a similar AI phenotype [28,29]. In health the secretory ameloblasts secrete a matrix that mineralises. This process requires movement of Ca^2+^ and associated ions across the enamel organ. Loss of FAM20C function prevents any meaningful enamel matrix being secreted with early enamel organ failure evident in Fam20c-null mice [28,29]. The presence of hypoplastic AI is probably one of the most distinctive features of loss of FAM20C function in individuals surviving into childhood and this was the main presenting feature in family 1. Recent mouse data indicate that amelogenesis failure is likely to be independent of the mechanisms causing the dentine defects and unrelated to alteration in FGF23 and phosphate homeostasis [36,37]. Instead, phosphorylation failure and down-regulation of enamel proteins such as ameloblastin (AMBM) and amelotin (AMTN) may be the key events.

In contrast to the severe and consistent negative impact on amelogenesis, the impact of loss of FAM20C function on dentine is more variable, although dentinogenesis was abnormal in both the reported families. Mantle dentine did not appear to be affected, which may reflect that biomineralisation at this stage is regulated by matrix vesicles [38,39]. The severity of crown and radicular dentine impairment varied between the families and between affected members of the same family. The absence of calcospherite fusion in the crown and radicular circumpulpal dentine was more severe in Family 2.

It is unknown whether the abnormal dentine primarily reflects loss of direct FAM20C function, such as altered phosphorylation of target proteins, or whether the abnormal phenotype is primarily a consequence of hypophosphataemia during dentinogenesis. The dentine non-collagenous phosphorylated proteins such as dentine sialoprotein (DSP), dentine phosphoryn (DPP) and dentine matrix protein 1 (DMP1) are essential for dentine mineralization. The affected dentine phenotype in Family 2 was remarkably similar to that observed in *Fam20c* null mice [28]. There were also similarities with the dentine observed in dentinogenesis imperfecta type III (DGIII) (OMIM #125500) due to mutations in *DSPP*, with comparable dentine changes observed in Dspp-null mice [40-42]. Furthermore, in *Dmp1* null mice dentine is also structurally abnormal as well as having a reduced thickness with an associated enlargement of the pulp chamber similar to that observed in Family 2 [43]. DMP also participates in phosphate homeostasis. A recent study reported that FAM20C supresses FGF23 production by enhancing DMP1 expression and that inactivation mutations in *FAM20C* cause FGF23 related hypophosphatemia by decreasing transcription of DMP1 [44]. It was not possible to measure the plasma FGF23 values of the families described in this study, although FGF23 has been persistently described as raised in some individuals with non-lethal Raine syndrome [16,18]. Inherited hypophosphataemic disorders are recognised to have interglobular dentine similar to that observed in Family 2 [45,46].

FAM20C is a paralogue of FAM20A and FAM20B. There are similarities between the oral phenotypes observed in individuals with either recessive mutations in *FAM20C* or *FAM20A* [47-49]. These may include poorly mineralised hypoplastic AI with incisal notching of permanent teeth that is most likely to be evident prior to eruption. Delayed tooth eruption, ectopic mineralisation of dental pulp or gingival tissue as well as variable gingival hyperplasia may also be present. The similar clinical features observed in the oral cavity with loss of FAM20A or FAM20C function indicates that the two proteins do not compensate for each other during amelogenesis and prevention of soft tissue mineralisation. It is the extra-oral developmental abnormalities associated with *FAM20C* mutations that facilitate distinction from individuals with *FAM20A* mutations who do not have bone abnormalities, but are at risk of nephrocalcinosis.

## Conclusions

We describe the first detailed oro-dental phenotype of two consanguineous families with recessive *FAM20C* mutations. This, together with the variable nature of the skeletal phenotype, broadens the understanding of the non-lethal clinical phenotype associated with *FAM20C* mutations. A profound failure of dental enamel formation leading to a distinctive hypoplastic AI in all teeth should alert clinicians to the possibility of *FAM20C* mutations.

## Additional file

Additional file 1: Table S1. Summary of bioinformatics analyses undertaken to predict the pathogenic nature of the missense mutations identified. **Table S2.** Primers designed using ExonPrimer to amplify the exons and surrounding intronic sequence of *FAM20C*. **Figure S1.** Conservation of the FAM20C P496 residue in orthologues. **Figure S2.** Oro-dental features of Family 1. **Figure S3.** Oro-dental features of Family 2.
