# Supplementary material for: Variability of systemic and oro-dental phenotype in two families with non-lethal Raine syndrome with FAM20C mutations
Source: BMC Med Genet. 2015 Feb 21;16:8. doi: 10.1186/s12881-015-0154-5 (PMC4422040; doi:10.1186/s12881-015-0154-5)
Supplement: Additional file 1: Table S1. — Summary of bioinformatics analyses undertaken to predict the pathogenic nature of the missense mutations identified. Table S2. Primers designed using ExonPrimer to amplify the exons and surrounding intronic sequence of FAM20C. Figure S1. Conservation of the FAM20C P496 residue in orthologues. Figure S2. Oro-dental features of Family 1. Figure S3. Oro-dental features of Family 2. [file 12881_2015_154_MOESM1_ESM.doc]

***Supplementary Data***

**Supplementary Table 1** - Summary of bioinformatics analyses undertaken to predict the pathogenic nature of the missense mutations identified. URLs: PolyPhen2, <http://genetics.bwh.harvard.edu/pph2/> ; Mutationtaster, <http://www.mutationtaster.org/> ; SIFT, <http://sift.jcvi.org/> ; Blosum62; PROVEAN, <http://provean.jcvi.org/> ; MutPred, <http://mutpred.mutdb.org/> .

‎

| **Mutation** | **PolyPhen2** | **MutationTaster** | **SIFT** | **Blosum62*** | **PROVEAN** | **MutPred** |
| --- | --- | --- | --- | --- | --- | --- |
| COL27A1  p.L124V | 0.911  Possibly Damaging | 0.999  Disease Causing | 0  Damaging | +1 | -1.924  Neutral | Deleterious Probability =  0.364 |
| ROBO3  p.T613I | 0.417  Benign | 0.811  Polymorphism | 0.19  Tolerated | -1 | -2.898  Deleterious | Deleterious Probability =  0.545 |
| KIRREL3  p.R336Q | 1.00  Probably Damaging | 0.999  Disease Causing | 0.04  Damaging | +1 | -1.677  Neutral | Deleterious Probability =  0.769 |
| FAM20C p.P496L | 1.000  Probably Damaging | 0.999  Disease Causing | 0  Damaging | -3 | -9.060  Deleterious | Deleterious Probability =  0.901 |

*Blosum62 scores range from +3 to -3 with negative scores being more likely to be damaging substitutions.

**Supplementary Table 2** – Primers designed using ExonPrimer to amplify the exons and surrounding intronic sequence of *FAM20C*. Primers used to amplify FAM20C cDNA (RT) were designed using Primer3.

| **Exon** | **F Primer (5’-3’)** | **R Primer (5’-3’)** |
| --- | --- | --- |
| 1-1 | GCACCGATGGACCTTGAC | GGTCGTGGGGTCTTAGGG |
| 1-2 | GGCCCAACAAGCACACG | GCCCCTGAACCTCTCTACAC |
| 2 | ATCTGCACTTGCTTGAACCC | GGCCTCCCAGACCTCTCTAA |
| 3 | GACCATGCCCAGAGGACC | AAAACACCCTGGGAGGAGAC |
| 4 | TGAGGAACCCAGCACGTC | AGGACGGCCTCACTCACC |
| 5 | CTTATTTGGAGGCAGGGAC | TCAGCACCCTGGTGTGGA |
| 6 | GGCCGTGAGACCACAGGT | AGCGGTCATCTCACACAGG |
| 7-8 | TGCCGCAGTGTTTCTCTTCT | CAGGTGGGCTGCAGGTAG |
| 9 | GTGTCGGGTACAGGCAGGT | GCTGTGGCCTCCTCTGTCT |
| 10 | TCCCTCTCACTTTCTCTCGC | TGTCCTATGAGACCTGGGGA |
| RT | CTGGCCCAACAAGCACAC | CCCGTAATTCTGGAAGGTCA |

**Supplementary Figure 1** – Conservation of the FAM20C P496 residue in orthologues. Conserved residues are highlighted. Human (NP_064608), Macaque (NP_001180642), Dog (XP_005621279), Cat (XP_003998439), Horse (XP_005598654), Cow (XP_614520), Rat (NP_001012238), Opossum (XP_001378055), Chicken (XP_414753) and Zebrafish (XP_001332042). Conservation of the P496 residue in Human FAM20A (NP_060035) and FAM20B (NP_055679) is also shown.

**P496L**

*

Human 480 RGFGKYSHDELSILVPLQQCCRIRKSTYLRL 511

Macaque 480 RGFGKYSHDELSILVPLQQCCRIRKSTYLRL 511

Dog 478 RGFGKYSHDELSILVPLQQCCRIRKSTYLRL 509

Cat 527 RGFGKYSHDELSILVPLQQCCRVRKSTYLRL 558

Horse 494 RGFGKYSHDELSILVPLQQCCRIRKSTHLRL 525

Cow 456 RGFGKHSHDELSILVPLQQCCRIRRSTYLRL 487

Rat 475 RGFGKYSHDELSILAPLHQCCRIRRSTYLRL 506

Opossum 487 RGFGKYSHDELSILVPLNQCCRIRKSTYLRL 518

Chicken 401 RGFGKYSHDELSILVPLNQCCRIRKSTYLRL 432

Zebrafish 455 RGFGKHSHDEMSILVPLTQCCRVKRSTYLRL 486

FAM20A 433 RGFGRHSHDEISILSPLSQCCMIKKKTLLHL 464

FAM20B 312 KSFGNPSLDERSILAPLYQCCIIRVSTWNRL 342

**Supplementary Figure 2 - Oro-dental features of family 1.** Clinical photograph of patient IV-4 (A) and patient IV-5 (B) showing alterations in the tooth shape, reduction of the enamel thickness and yellow and brown discoloration. Note the rough and pitted surface of enamel. Incisal notches were observed in the central incisors of both patients. In patient IV-4 radiographic examination showed no differential radiodensity between enamel and dentine layers. Apical and periodontal radiolucencies suggestive of apical and periodontal abscesses were observed.

**
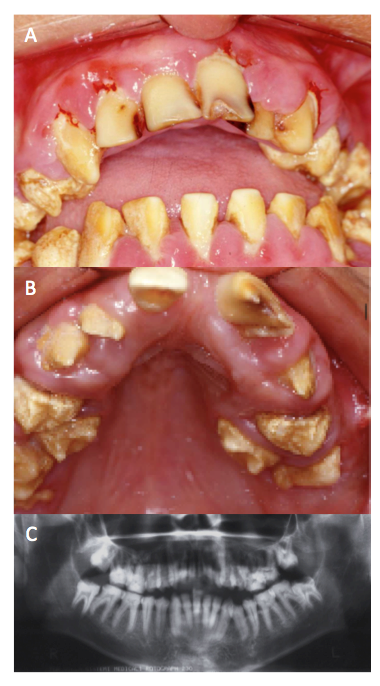
**

**Supplementary Figure 3 - Oro-dental features of Family 2 – patient VI-2.** The patient presented with permanent erupted teeth with yellow discoloration, hypocalcified and hypoplastic enamel (A & B). Severe delay in permanent tooth eruption were also observed. Radiographic analysis revealed an absence of density differences between enamel and dentin, incomplete root formation and enlarged pulp chambers (C). Apical radiolucencies associated with unerupted teeth can be observed.

**
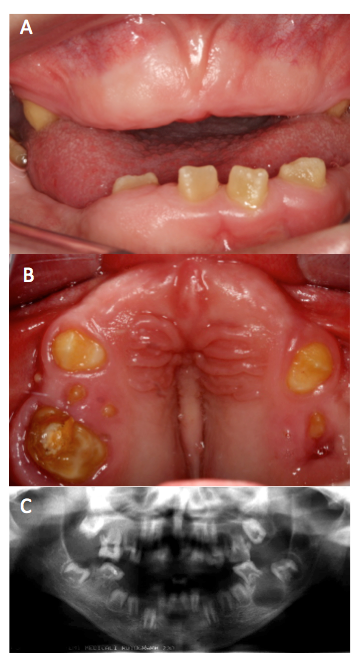
**

**Supplementary References**

1 Adzhubei, I.A., Schmidt, S., Peshkin, L., Ramensky, V.E., Gerasimova, A., Bork, P., Kondrashov, A.S. and Sunyaev, S.R. (2010) A method and server for predicting damaging missense mutations. *Nature methods*, **7**, 248-249.

2 Schwarz, J.M., Rodelsperger, C., Schuelke, M. and Seelow, D. (2010) MutationTaster evaluates disease-causing potential of sequence alterations. *Nature methods*, **7**, 575-576.

3 Ng, P.C. and Henikoff, S. (2003) SIFT: Predicting amino acid changes that affect protein function. *Nucleic acids research*, **31**, 3812-3814.

4 Henikoff, S. and Henikoff, J.G. (1993) Performance evaluation of amino acid substitution matrices. *Proteins*, **17**, 49-61.

5 Choi, Y., Sims, G.E., Murphy, S., Miller, J.R. and Chan, A.P. (2012) Predicting the functional effect of amino acid substitutions and indels. *PloS one*, **7**, e46688.

6 Li, B., Krishnan, V.G., Mort, M.E., Xin, F., Kamati, K.K., Cooper, D.N., Mooney, S.D. and Radivojac, P. (2009) Automated inference of molecular mechanisms of disease from amino acid substitutions. *Bioinformatics*, **25**, 2744-2750.
